# Supplementary material for: Massive Southern Ocean phytoplankton bloom fed by iron of possible hydrothermal origin
Source: Nat Commun. 2021 Feb 22;12:1211. doi: 10.1038/s41467-021-21339-5 (PMC7900241; doi:10.1038/s41467-021-21339-5)
Supplement: Supplementary file 1 — Supplementary Information [file 41467_2021_21339_MOESM1_ESM.pdf]

Supplementary Information:

Massive Southern Ocean phytoplankton bloom fed by iron of possible hydrothermal origin

Schine et al.

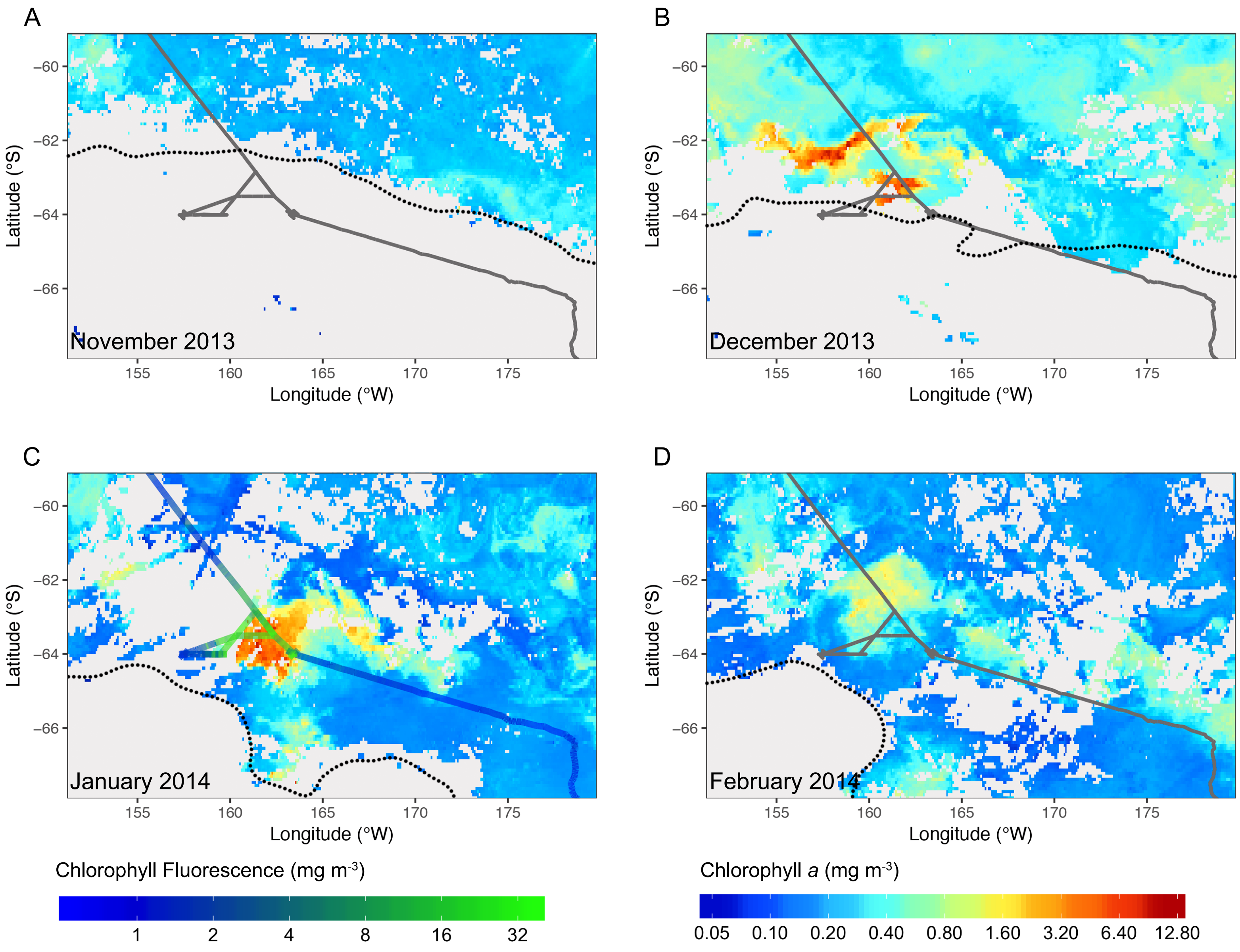

Supplementary Figure 1. Position of the bloom from November 2013 through February 2014. Maps of mean monthly Chl *a* and the position of the sea ice edge from (a) November 2013, (b) December 2013, (c) January 2014, and (d) February 2014. Cruise track is shown as a dark gray line for (a), (b), and (d), and as a green/blue line for (c), when line color is indicative of underway fluorescence intensity as indicated by the color bar. Mean position of the sea ice edge is indicated by the dotted line. Gray pixels indicate an absence of data for the entire month. Gray pixels north of the sea ice edge line indicate cloud cover.

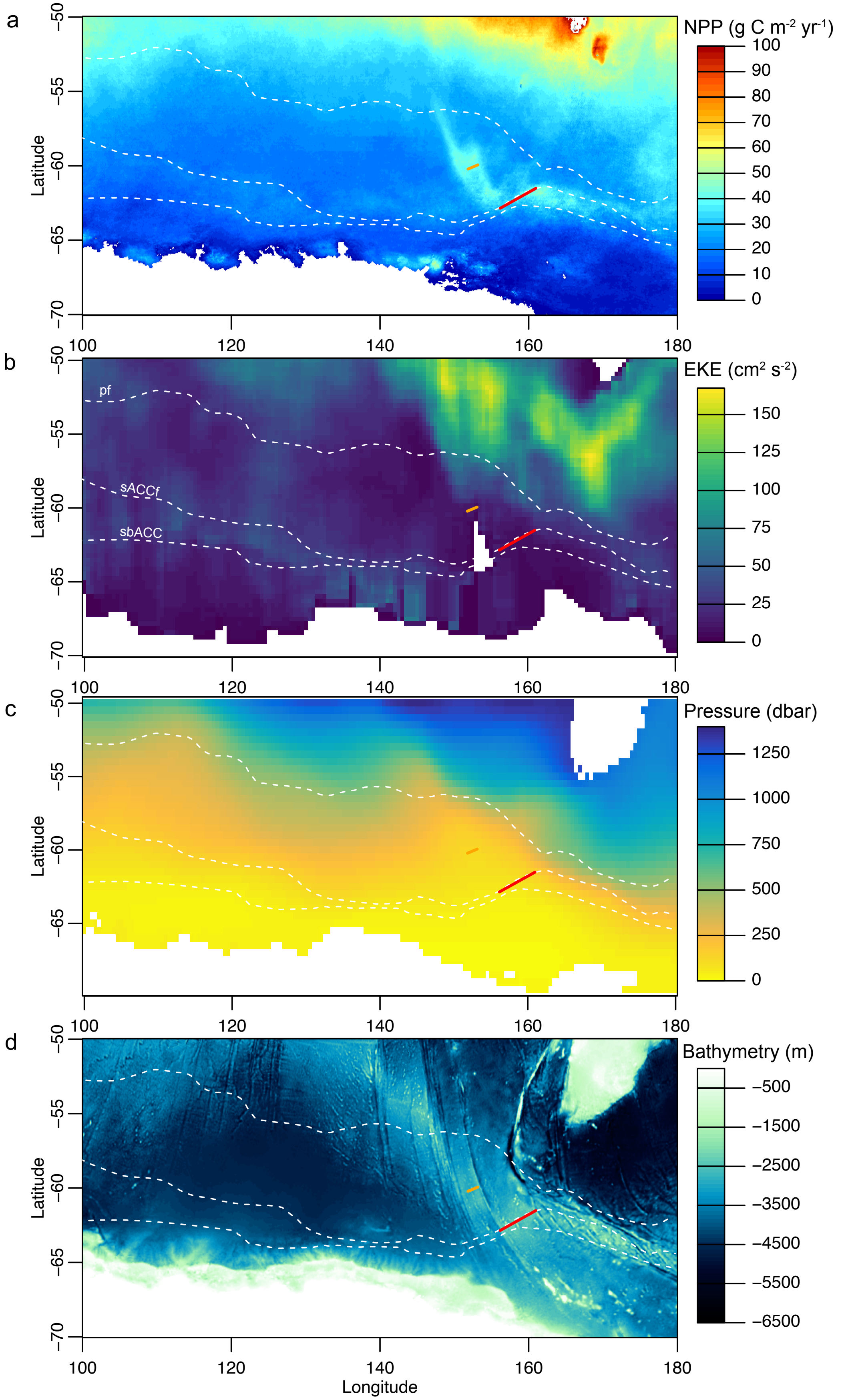

Supplementary Figure 2. Position of the bloom relative to factors that promote upwelling. Maps of (a) NPP climatology, (b) deep eddy kinetic energy (EKE), (c) pressure at isopycnal $\sigma_\theta = 27.49$, and (d) bathymetry. The position of KR1 and KR2 are shown by the red line and the orange line, respectively. The position of the polar front, the southern Antarctic Circumpolar Current (ACC) front (sACCf), and the southern boundary of the ACC (sbACC), from north to south, are indicated by the dashed white lines. Bathymetry data are from https://www.ngdc.noaa.gov/mgg/global/.

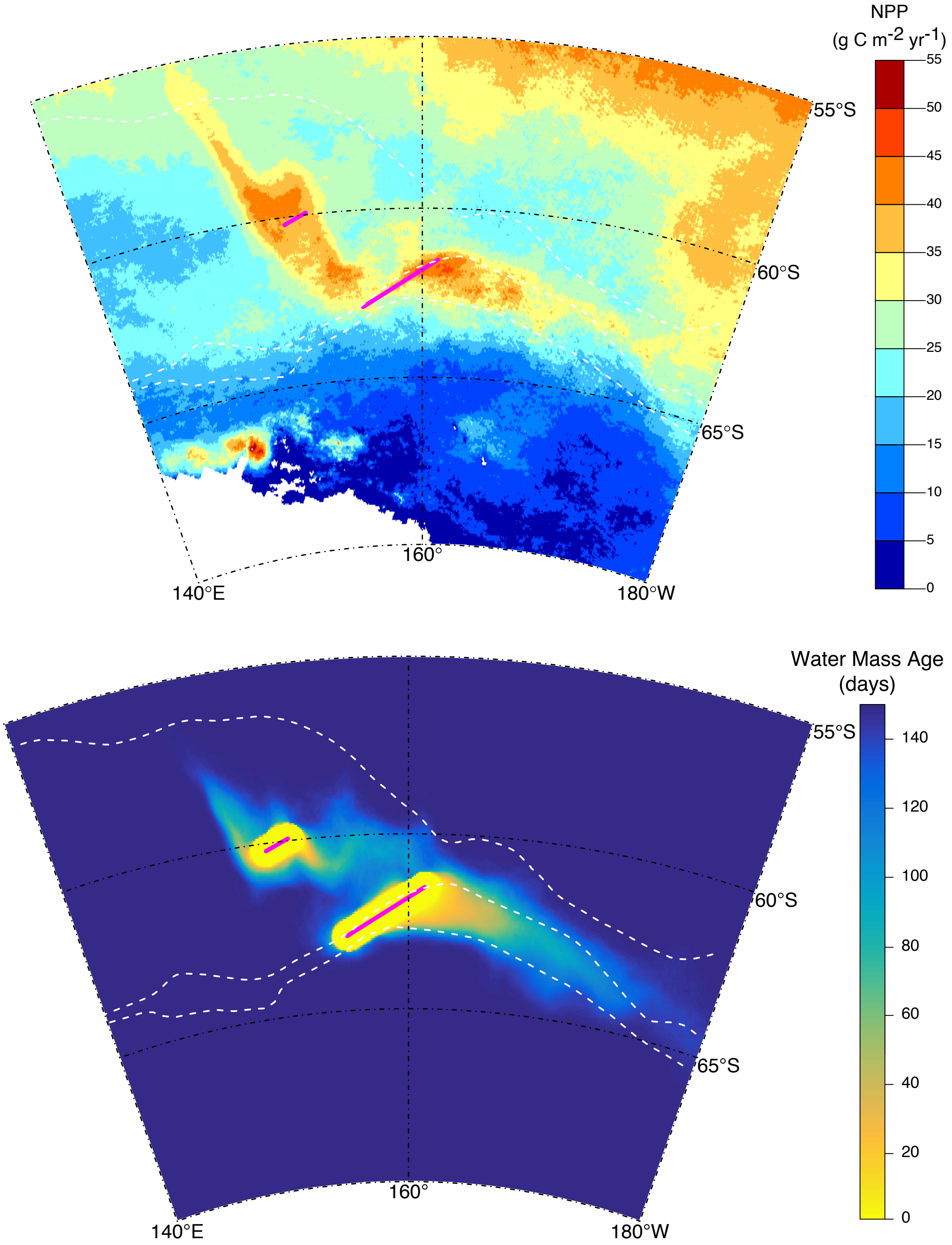

Supplementary Figure 3. Position of the bloom relative to the position of the Lagrangian plume generated by releasing particles at the surface above the vents. Maps of (a) Net Primary Production (NPP) climatology (1997-2019) and the (b) climatology of the Lagrangian plume stemming from the surface directly above the position of the hydrothermal vents. The Lagrangian plume shows the distance in time of water masses to the vents. Vent positions are indicated by the pink lines. The positions of the polar front, the southern ACC front (sACCf), and the southern boundary of the ACC (sbACC) from north to south, respectively[1], are shown by the white dashed lines.

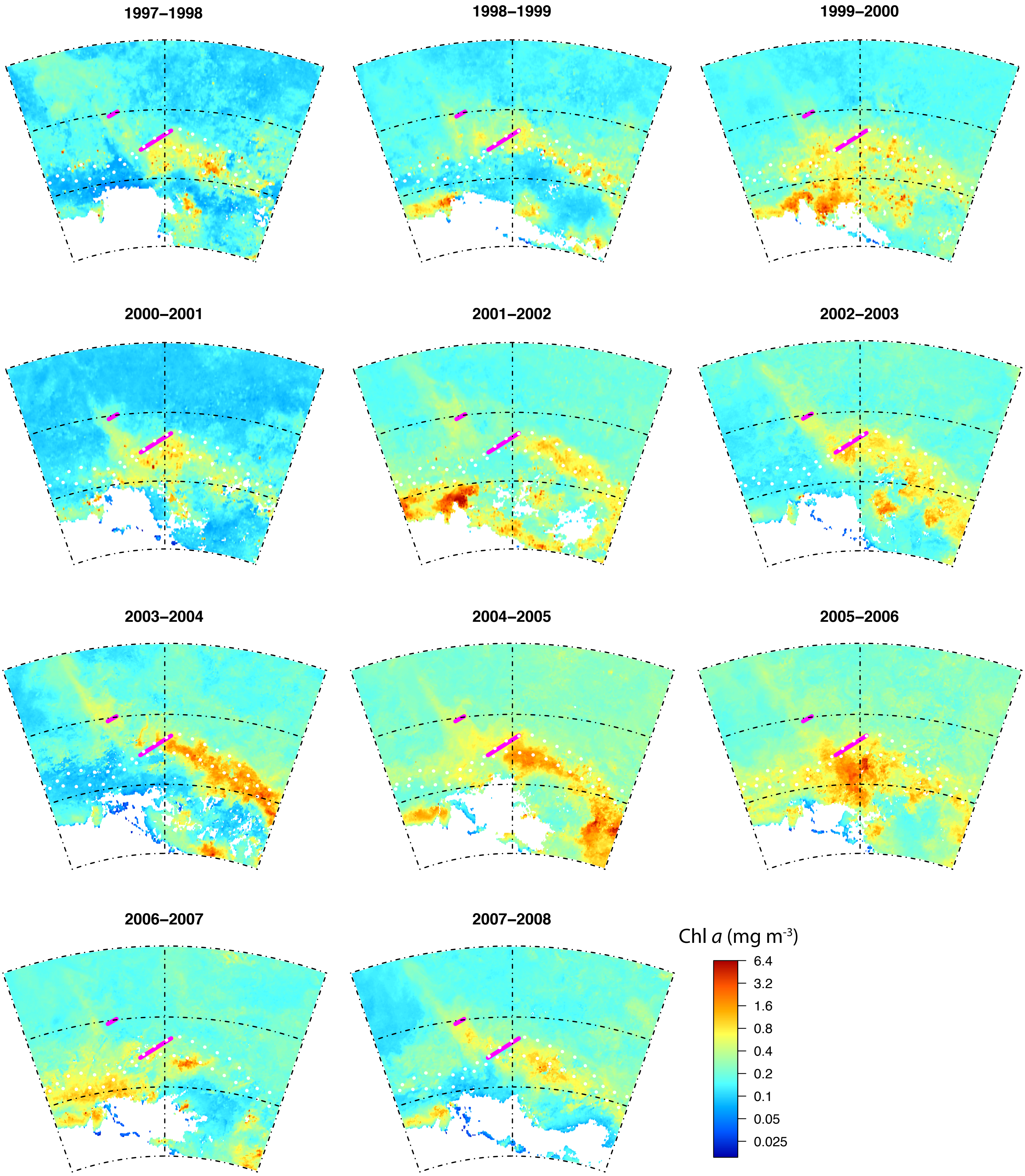

Supplementary Figure 4. Mean satellite Chl *a* concentration from October to March from 1997 through 2019. The positions of KR1 and KR2 are shown by the pink lines and the position of the southern Antarctic Circumpolar Current (ACC) front (sACCf) and southern boundary of the ACC (sbACC) are shown as white dotted lines from north to south, respectively.

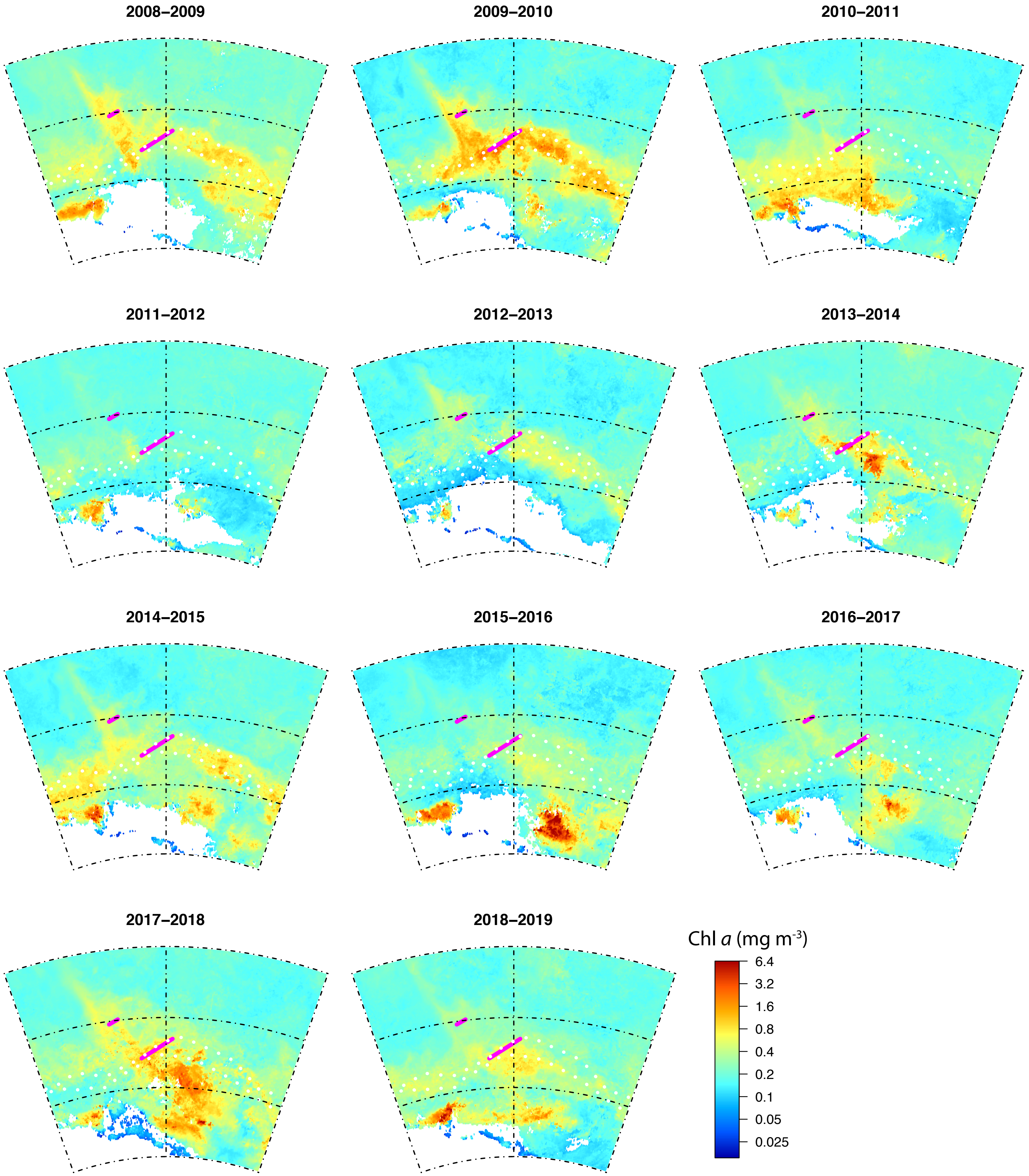

Supplementary Figure 4 (continued). Mean satellite Chl *a* concentration from October to March from 1997 through 2019. The positions of KR1 and KR2 are shown by the pink lines and the position of the southern Antarctic Circumpolar Current (ACC) front (sACCf) and southern boundary of the ACC (sbACC) are shown as white dotted lines from north to south, respectively.

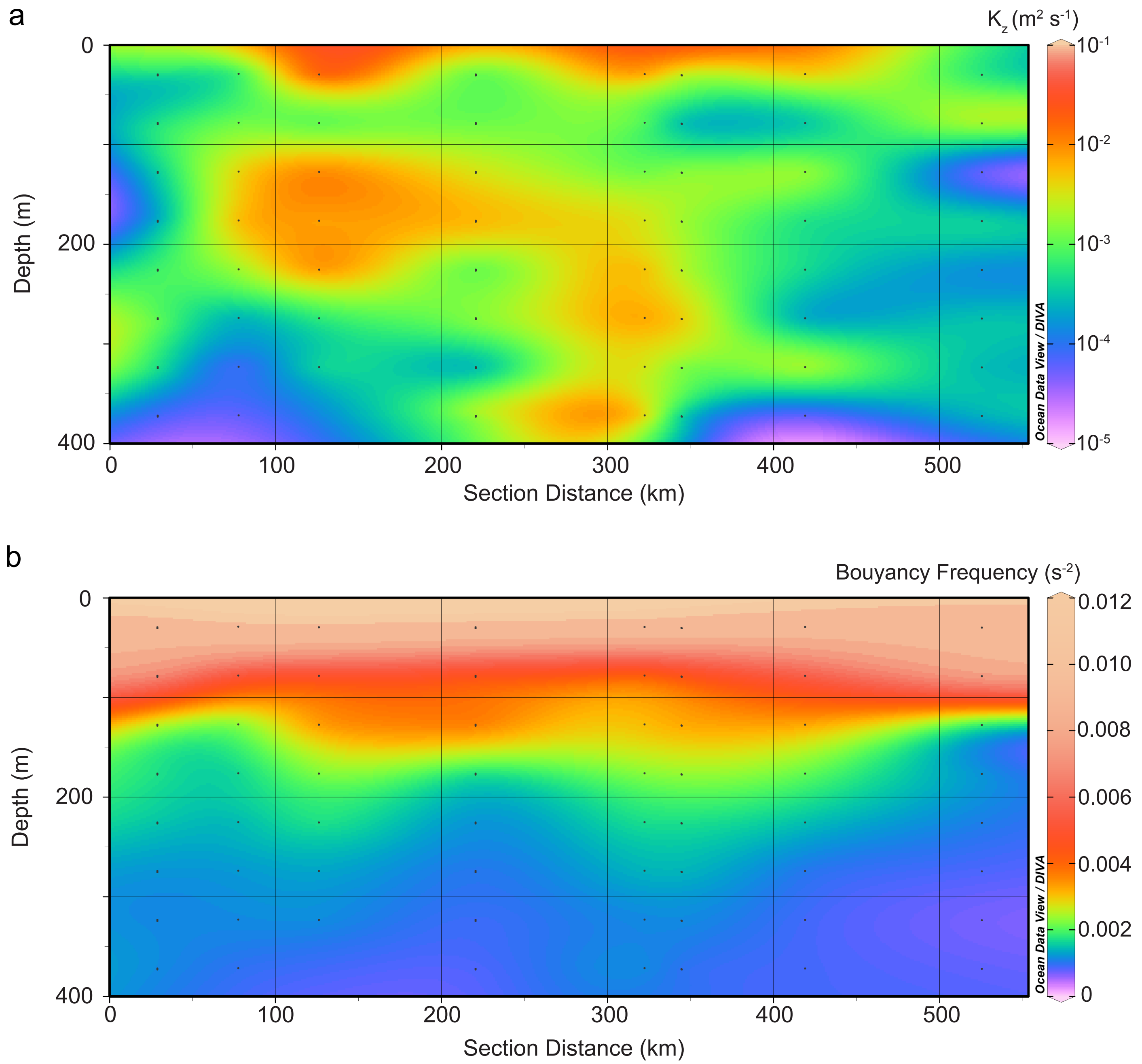

Supplementary Figure 5. Elevated vertical diffusivity (K_z) coincides with elevated stratification. Sections of (a) log-transformed vertical diffusivity and (b) buoyancy frequency. A map of the portion of the cruise shown in these sections and the stations that corresponds with the stations here is shown in Figure 1d. Station 122 is on the map but not included in the section.

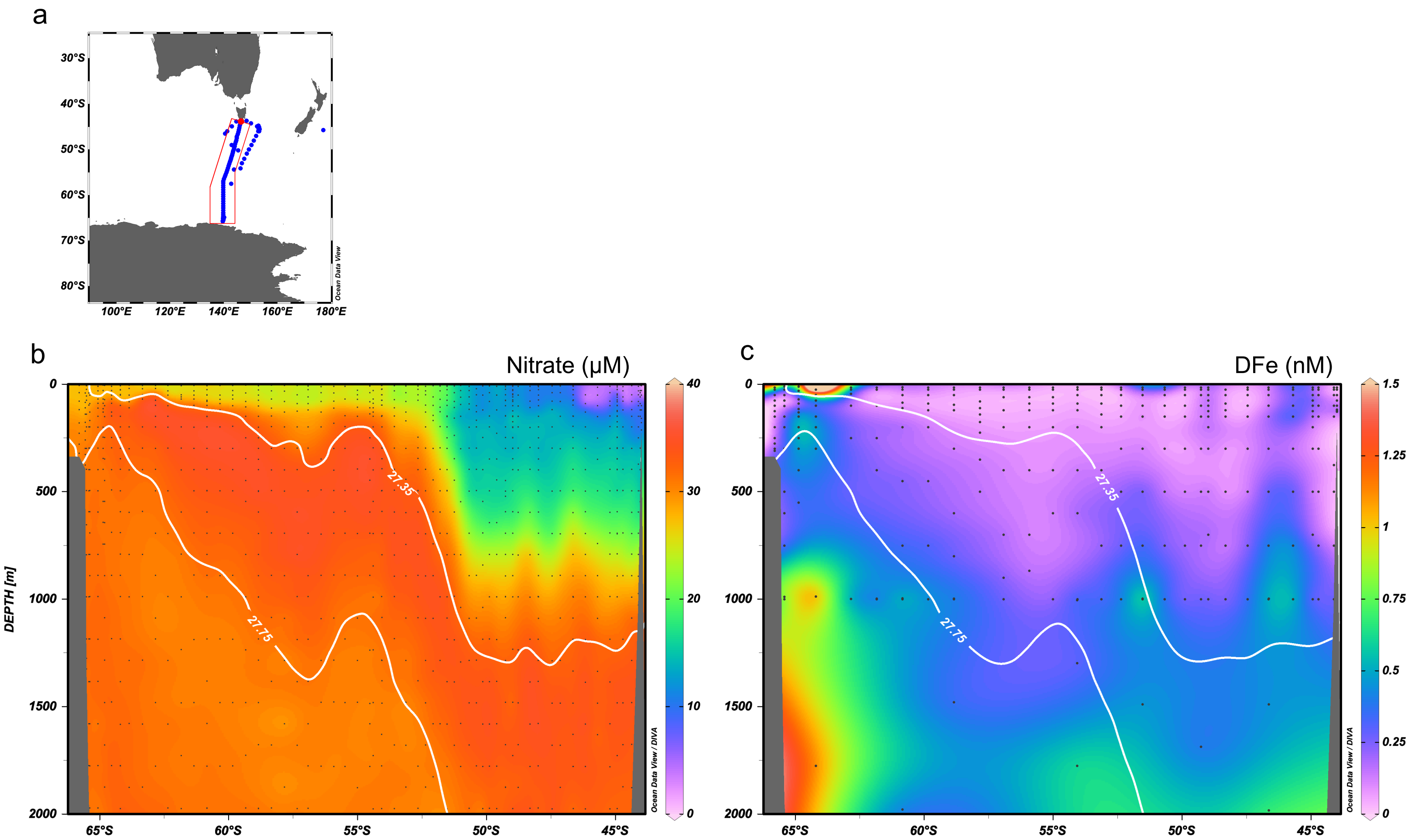

Supplementary Figure 6. Nitrate and dissolved iron (DFe) concentrations in Upper Circumpolar Deep Water (UCDW) in the Pacific sector of the Southern Ocean. (a) Map of GEOTRACES transect from the GIPY06 cruise from Tasmania to Antarctica in 2008, with section showing (b) nitrate and (c) DFe concentrations. The white contours in (b) and (c) indicate the bounds of the upper and lower density anomaly (referenced to the surface) associated with UCDW (27.35-27.75 kg m^{-3})[1,2]. GEOTRACES Intermediate Data Product 2017 (Version 2) is available at https://www.bodc.ac.uk/geotraces/data/idp2017/.

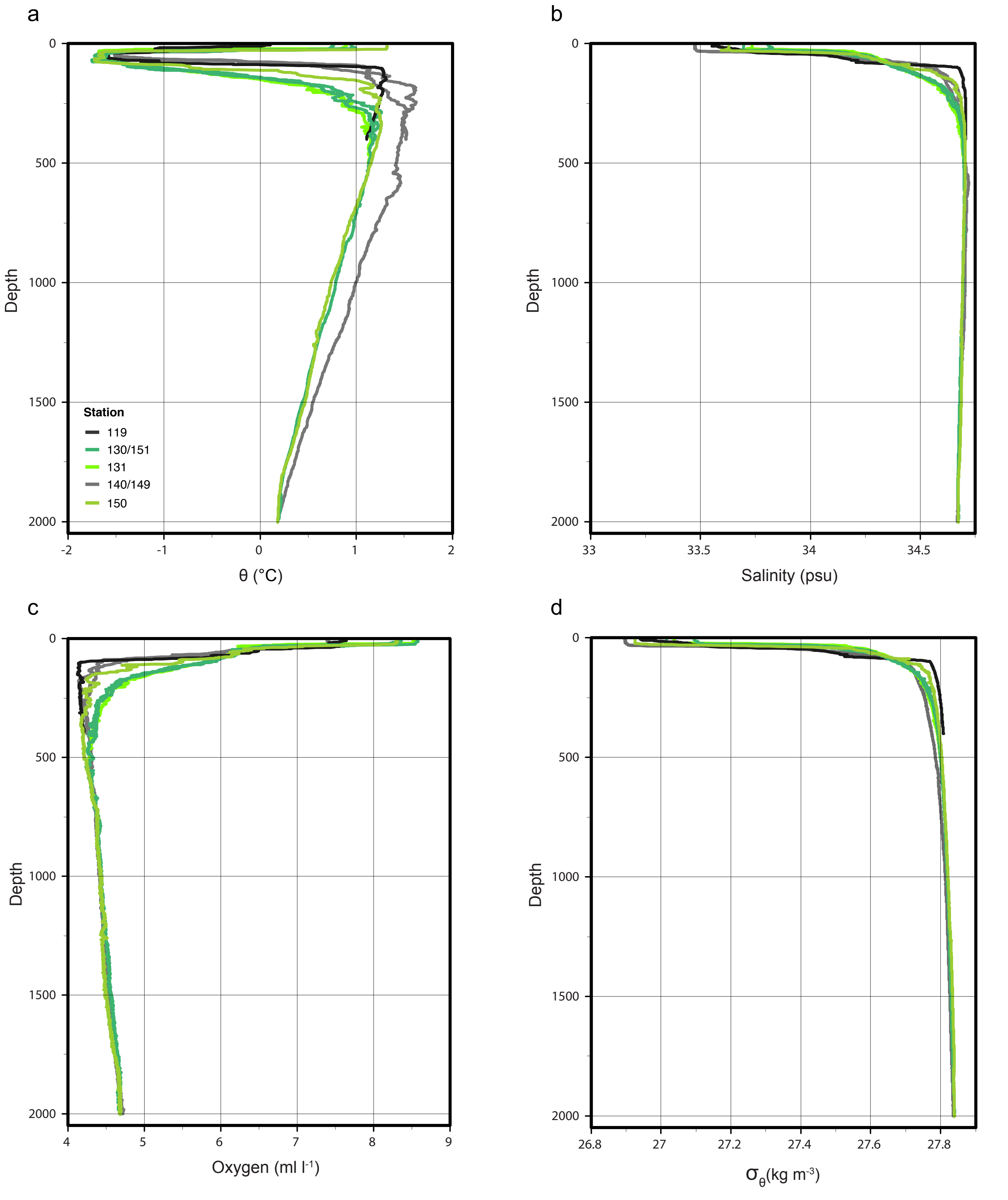

Supplementary Figure 7. Water mass properties at bloom stations versus non-bloom stations. Depth profiles of (a) potential temperature, (b) salinity, (c) oxygen, and (d) potential density for stations inside the bloom (green lines) and stations outside the bloom (gray lines).

Supplementary Methods

Vertical turbulent eddy diffusivity estimates using CTD-data

CTD samples were obtained at a rate of 24 Hz. The average lowering speed was 0.4 m s^{-1}, so that measurements are made about every 0.017 m. During post-processing using SBE-software, potential surface-wave-influences, that may reverse direction of motion of the package, are filtered out by restricting to a CTD-speed of >|0.25| m s^{-1}, whereby direction changes are removed. Corrections for thermal inertia of conductivity cells are applied and the data are transferred into 0.33 m vertical bins, thereby averaging some 20 data points. This associates with the typical turbulent overturn-scale of 0.4 m found resolvable using CTD-data by Stansfield et al.[3]. Ideally, it is preferred to use temperature-only data, under the condition of a tight T-S relationship, as a tracer for density to compute turbulence parameter estimates. This holds not only in lakes, as in the original paper by Thorpe[4], but also in the ocean, as CTD-computed density (anomaly) data are 3-10 times noisier than density from temperature alone[3,5]. However, for polar regions where many salinity compensated intrusions exist, e.g. due to ice effects, one has to use CTD's density data for turbulence parameter estimates[6].

The vertical turbulent eddy diffusivity (K_z) was estimated by calculating the 'Thorpe scale' (d_T) using the 0.33 m binned CTD-sigma_theta (density anomaly referenced to the surface) data. The parameter d_T is a vertical length scale of turbulent mixing in a stratified flow[4]. It is obtained by rearranging an observed potential density profile, which may contain inversions associated with turbulent overturns, into a stable profile without inversions. The vertical displacement necessary to generate the stable profile is the 'Thorpe displacement'. A certain threshold applies to disregard apparent displacements associated with instrumental noise. Here, a threshold of 0.0005 kg m^{-3} is used, as in Gargett and Garner[6]. These authors also propose to test for (a)symmetric distribution of positive and negative displacements to rule out spikes. In the present data, mostly obtained in good weather conditions, nearly all displacements passed this test, implying effective removal of spikes by the post-processing.

Defining d_T as the root mean square of the Thorpe displacements within each turbulent overturn, the eddy diffusivity (m^2 s^{-1}) is obtained as:

$$K_z = 0.128\, d_T^2\, N \qquad (2)$$

where N denotes the buoyancy frequency and the constant 0.128 is derived from an empirical relation with the Ozmidov scale, the largest overturn scale in stratified waters, using a constant mixing efficiency of 0.2, which is typical for shear-induced turbulence[7]. The method of overturn displacements provides a reasonably adequate estimate of K_z and the turbulence dissipation rate to within a factor of two, as has been established after comparison with independent estimates using free-falling microstructure data[8].

The raw K_z(z) profiles were averaged in 50 m vertical bins, similar to the largest displacement observed, over which mean K_z values were calculated.

References

1 Orsi, A. H., Whitworth, T. & Nowlin, W. D. On the meridional extent and fronts of the Antarctic Circumpolar Current. *Deep-Sea Research I* **42**, 641-673, doi:10.1016/0967-0637(95)00021-w (1995).

2 Hiscock, M. R. *et al.* Primary productivity and its regulation in the Pacific Sector of the Southern Ocean. *Deep-Sea Research II* **50**, 533-558 (2003).

3 Stansfield, K., Garrett, C. & Dewey, R. The probability distribution of the Thorpe displacement within overturns in Juan de Fuca Strait. *J. Phys. Oceanogr*. **31**, 3421-3434 (2001).

4 Thorpe, S.A. Turbulence and mixing in a Scottish Loch. *Philos. Trans. Roy. Soc. Lond. A* **286**, 125-181 (1977).

5 van Haren, H. & Gostiaux, L. Characterizing turbulent overturns in CTD-data. *Dyn. Atmos. Oceans* **66**, 58-76 (2014).

6 Gargett, A. & Garner, T. Determining Thorpe scales from ship-lowered CTD density profiles. *J. Atmos. Ocean. Tech.* **25**, 1657-1670 (2008).

7 Dillon, T.M. Vertical overturns: a comparison of Thorpe and Ozmidov length scales. *J. Geophys. Res.* **87**, 9601-9613 (1982).

8 Hosegood, P., van Haren, H., & Veth, C. Mixing within the interior of the Faeroe-Shetland Channel. *J. Mar. Res.* 63, 529-561 (2005).
