# Supplementary material for: Massive Southern Ocean phytoplankton bloom fed by iron of possible hydrothermal origin
Source: Nat Commun. 2021 Feb 22;12:1211. doi: 10.1038/s41467-021-21339-5 (PMC7900241; doi:10.1038/s41467-021-21339-5)
Supplement: Supplementary file 3 — Reporting Summary [file 41467_2021_21339_MOESM3_ESM.pdf]

Corresponding author(s): Casey M.S. Schine
Last updated by author(s): Jan 20, 2021

Reporting Summary

Nature Research wishes to improve the reproducibility of the work that we publish. This form provides structure for consistency and transparency in reporting. For further information on Nature Research policies, see our Editorial Policies and the Editorial Policy Checklist.

Statistics

For all statistical analyses, confirm that the following items are present in the figure legend, table legend, main text, or Methods section.

n/a	Confirmed	
☒	☐	The exact sample size (*n*) for each experimental group/condition, given as a discrete number and unit of measurement
☐	☒	A statement on whether measurements were taken from distinct samples or whether the same sample was measured repeatedly
☐	☒	The statistical test(s) used AND whether they are one- or two-sided<br>*Only common tests should be described solely by name; describe more complex techniques in the Methods section.*
☒	☐	A description of all covariates tested
☐	☒	A description of any assumptions or corrections, such as tests of normality and adjustment for multiple comparisons
☐	☒	A full description of the statistical parameters including central tendency (e.g. means) or other basic estimates (e.g. regression coefficient) AND variation (e.g. standard deviation) or associated estimates of uncertainty (e.g. confidence intervals)
☐	☒	For null hypothesis testing, the test statistic (e.g. *F*, *t*, *r*) with confidence intervals, effect sizes, degrees of freedom and *P* value noted<br>*Give P values as exact values whenever suitable.*
☒	☐	For Bayesian analysis, information on the choice of priors and Markov chain Monte Carlo settings
☒	☐	For hierarchical and complex designs, identification of the appropriate level for tests and full reporting of outcomes
☒	☐	Estimates of effect sizes (e.g. Cohen's *d*, Pearson's *r*), indicating how they were calculated

Our web collection on statistics for biologists contains articles on many of the points above.

Software and code

Policy information about availability of computer code

Data collection	Sea Bird 911 plus CTD package was used for collecting water column measurement data.
Data analysis	Analyses of the results were done with R version 3.6. 2 (R Project for Statistical Computing) and Sea DAS version 7.5.3 (NASA). CHEMTAX analysis package (version 1.95) was used to assess phytoplankton class abundance.

For manuscripts utilizing custom algorithms or software that are central to the research but not yet described in published literature, software must be made available to editors and reviewers. We strongly encourage code deposition in a community repository (e.g. GitHub). See the Nature Research guidelines for submitting code & software for further information.

Data

Policy information about availability of data

All manuscripts must include a data availability statement. This statement should provide the following information, where applicable:
- Accession codes, unique identifiers, or web links for publicly available datasets
- A list of figures that have associated raw data
- A description of any restrictions on data availability

The satellite data that support these findings are freely available and may be downloaded from the links provided here: MODIS/Aqua Chl a images are available from https://oceancolor.gsfc.nasa.gov/, SSM/I sea ice concentration data are available from http://www.nsidc.org/data/g02202, SSALTO/DUACS global gridded sea surface height (product id SEALEVEL_GLO_PHY_L4_REP_OBSERVATIONS_008_047) is available from https://marine.copernicus.eu, the ANDRO dataset is available from www.coriolis.eu.org/Data-Products/Products/ANDRO, and the MIMOC dataset is available from www.pmel.noaa.gov/mimoc/. The cruise data that support these findings are available at the Stanford Digital Depository (permanent URL: https://purl.stanford.edu/sn954dk6470).

Field-specific reporting

Please select the one below that is the best fit for your research. If you are not sure, read the appropriate sections before making your selection.

☐ Life sciences ☐ Behavioural & social sciences ☒ Ecological, evolutionary & environmental sciences

For a reference copy of the document with all sections, see nature.com/documents/nr-reporting-summary-flat.pdf

Ecological, evolutionary & environmental sciences study design

All studies must disclose on these points even when the disclosure is negative.

Study description	Sampling targeted a patch of high chlorophyll surface waters seen in daily satellite images. We targeted this patch during our research cruise sampling stations both inside and outside the patch.
Research sample	Discrete water samples were collected during each cast using both a trace-metal clean CTD-rosette package (TMC-CTD), and a conventional CTD-rosette package. For biological sampling, water was collected at depths of 10, 25, 50, 75, and 100 m, and an additional six to seven depths between 100 and 400 m. Water samples were collected for dissolved Fe, macronutrients (nitrate, nitrite, phosphate, and silicate), Chl a, particulate organic carbon, phytoplankton pigments, and simulated in situ primary production. Additionally, for the three casts conducted down to 2000 m, we collected water every 200 m below 400 m with the TMC-CTD-rosette for DFe analysis
Sampling strategy	o Our sampling strategy was to sample as many stations as possible both inside and outside the bloom in the time available to us based on ship availability and favorable weather conditions. Analytical replicated were collected for chemical and biological measurements, but as it is impossible to sample the same water parcel twice our sample size for each depth at each station can only ever be 1.
Data collection	Data was collected by members of the science team including the authors on this paper. We collected water samples with the CTD rosette and measured water properties using instruments mounted on the rosette frame as described in the Methods section.
Timing and spatial scale	Timing of our sampling was dictated by the timing of our transit fro the Ross Sea to Hobart, Tasmania as well as inclement weather in the region which cut our sampling short and forced us to leave the region slightly early. The spatial scale of our sampling was dictated by the size of the bloom relative to the amount of time available to sample enough stations to adequately capture the variability inside and outside the bloom.
Data exclusions	Station 135 was excluded from linear regression analysis of DFe flux, vertical diffusivity, and DFe vertical gradient vs. integrated Chl a, because it had an anomalously high vertical diffusivity that was more than two standard deviations above the mean.
Reproducibility	We did not conduct any experiments, but rather sampled the natural variability in our study region. Analytical replicates were taken for both chemical and biological measurements.
Randomization	Randomization is not appropriate for oceanographic survey measurements.
Blinding	Blinding is not appropriate for oceanographic survey measurements.

Did the study involve field work? ☒ Yes ☐ No

Field work, collection and transport

Field conditions	Field work was conducted on board the RVIB Nathaniel B. Palmer, water temperature ranged from -1 to 3 degrees Celsius, and air temperatures from -10 to 2.5 degrees Celsius.
Location	Sampling location was ~162 degrees S in water depths greater than 2000 m.
Access & import/export	Habitat sampled was in international waters and did not require a permit for access. No permits were required for the import/export of samples.
Disturbance	Sampling was conducted using standard oceanographic practices which result in virtually no disturbance of the environment.

Reporting for specific materials, systems and methods

We require information from authors about some types of materials, experimental systems and methods used in many studies. Here, indicate whether each material, system or method listed is relevant to your study. If you are not sure if a list item applies to your research, read the appropriate section before selecting a response.

Materials & experimental systems

n/a	Involved in the study
☒	☐ Antibodies
☒	☐ Eukaryotic cell lines
☒	☐ Palaeontology and archaeology
☒	☐ Animals and other organisms
☒	☐ Human research participants
☒	☐ Clinical data
☒	☐ Dual use research of concern

Methods

n/a	Involved in the study
☒	☐ ChIP-seq
☒	☐ Flow cytometry
☒	☐ MRI-based neuroimaging
